# Hardwired survival algorithms: RelBE1 and MadR1 couple carbon state to metabolic persistence in *Mycobacterium tuberculosis*

**DOI:** 10.3389/fmicb.2026.1863008

**Published:** 2026-08-10

**Authors:** Richard A. Slayden, Jason E. Cummings, Clinton C. Dawson

**Affiliations:** Department of Microbiology, Immunology, and Pathology, Colorado State University, Fort Collins, CO, United States

**Keywords:** carbon metabolism, host adaptation, MadR1, metabolic regulation, *Mycobacterium tuberculosis*, persistence, RelBE1, toxin-antitoxin systems

## Abstract

*Mycobacterium tuberculosis* (*Mtb*) survives host-imposed stress through dynamic metabolic adaptation and growth modulation. Toxin–antitoxin (TA) systems and cell cycle regulators, including RelBE1 and MadR1, are increasingly recognized as contributors to these processes, yet their physiological roles remain unclear. In this perspective, we propose that these systems function as genomically encoded metabolic control modules that couple carbon source availability to persistence. The *relBE1* loci, encoded adjacent to the α-ketoglutarate decarboxylase gene (*kgd*), and the cell division regulator, *madR1*, co-localized with the pyruvate dehydrogenase component gene (*dlaT*), are transcriptionally linked to key nodes of the tricarboxylic acid (TCA) cycle. Under lipid-rich, host-relevant conditions, these modules may coordinate changes in TCA flux, redox balance, and resource allocation. We suggest that RelBE1 modulates translation in a carbon-state-dependent manner, while MadR1 integrates metabolic signals with growth control. Together, these systems support a regulatory architecture that links environmental sensing to metabolic reprogramming and persistence. This framework reframes TA systems as metabolic integrators and identifies new regulatory components involved in survival during chronic infection.

## Introduction

**FIGURE 1:**
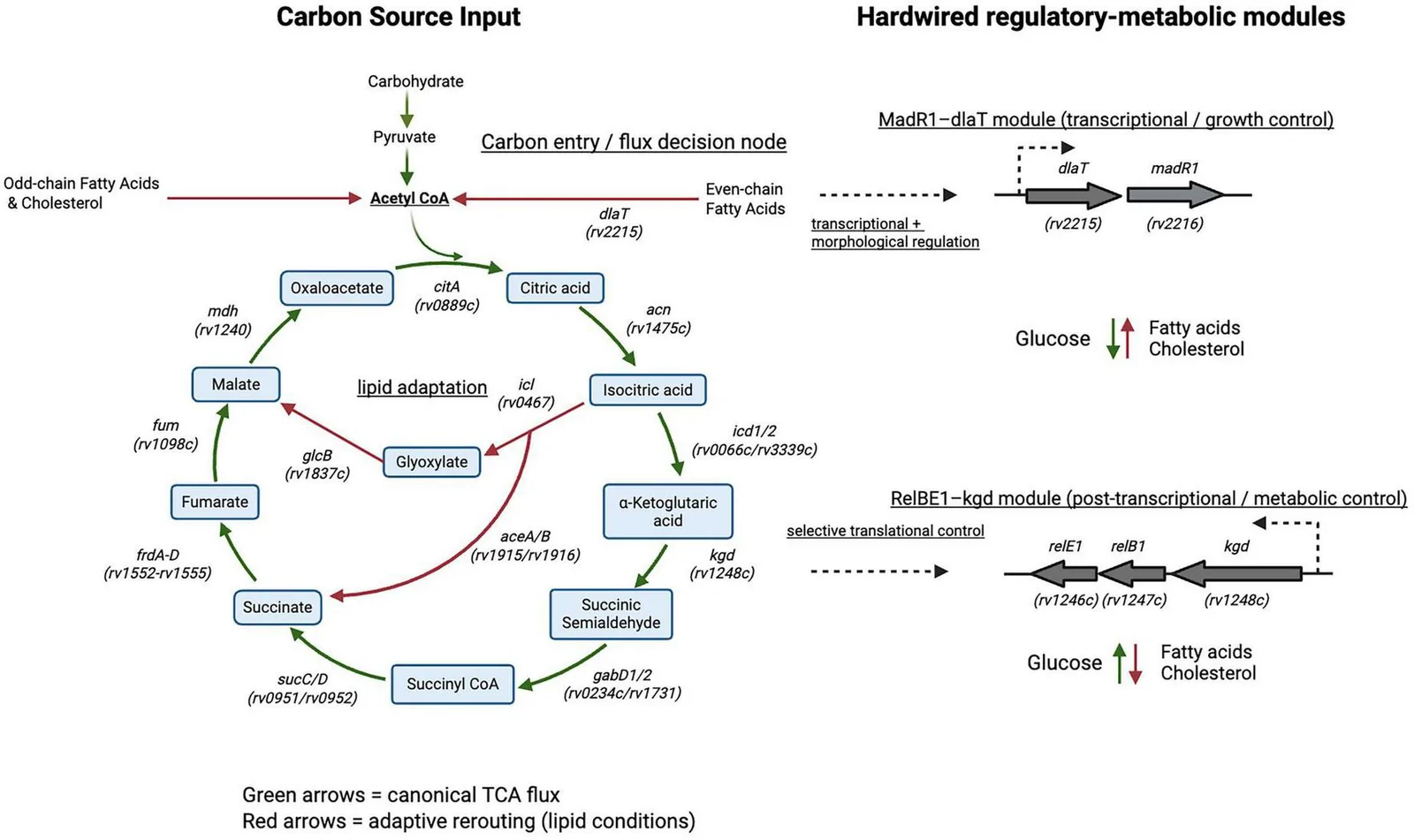
Carbon-source-dependent metabolic routing and regulatory coupling in *Mycobacterium tuberculosis*. This integrated schematic illustrates how carbon source availability is coupled to central metabolism and genomically encoded regulatory modules in *M. tuberculosis*. Carbohydrates, even-chain fatty acids, and odd-chain fatty acids/cholesterol converge at acetyl-CoA, which functions as a major carbon entry point into central metabolism. From this node, carbon is distributed through the tricarboxylic acid (TCA) cycle or redirected into adaptive pathways depending on nutrient conditions. Green arrows denote canonical TCA cycle flux under carbohydrate-replete conditions, whereas red arrows indicate adaptive rerouting associated with lipid-rich, host-relevant environments, including engagement of the glyoxylate shunt. Genomically coupled regulatory–metabolic modules are shown on the right. The *madR1–dlaT* locus links a SulA-like cell division regulator (MadR1; Rv2216) with the E2 component of pyruvate dehydrogenase (DlaT; Rv2215), positioning MadR1 to coordinate transcriptional and morphological programs with carbon entry through acetyl-CoA. In contrast, the *relBE1–kgd* locus couples a type II toxin–antitoxin system (RelB1–RelE1; Rv1246c–Rv1247c) with α-ketoglutarate decarboxylase (Kgd; Rv1248c), a key enzyme in alternative TCA routing. RelBE1 is proposed to act at the post-transcriptional level through conditional translational modulation, refining metabolic adaptation in response to carbon flux. Dashed arrows indicate bidirectional coupling between metabolic state and regulatory module activity, reflecting carbon-state-dependent activation and feedback modulation of metabolic flux. Under host-relevant conditions characterized by decreased glucose and increased fatty acids and cholesterol, coordinated engagement of MadR1 and RelBE1 promotes metabolic reprogramming, redox balance, and persistence-associated physiology. Together, these features support a model in which regulatory–metabolic modules function as carbon-state-responsive control elements embedded within central metabolism.

Tuberculosis (TB), caused by *Mycobacterium tuberculosis* (*Mtb*), remains a leading cause of infectious mortality worldwide (World Health Organization, 2025). A defining feature of *Mtb* pathogenesis is its ability to adapt to diverse and dynamic host environments, including nutrient limitation, acidic stress, and immune pressure. Central to this adaptability is the capacity to reprogram metabolism in response to environmental cues, enabling transitions between replicative and non-replicative states during infection (Ehrt and Rhee, 2013). *Mtb* survival *in vivo* depends on metabolic flexibility, particularly the utilization of host-derived carbon sources such as fatty acids and cholesterol (Chang and Guan, 2021; Samuels et al., 2022). These substrates feed into the tricarboxylic acid (TCA) cycle and associated bypass pathways, including the glyoxylate and methylcitrate cycles, which sustain energy production when carbohydrates are limited (Figure 1; Lee et al., 2013). While the biochemical pathways of lipid catabolism are increasingly well defined, the regulatory mechanisms coordinating these metabolic transitions with adaptive regulation and cell cycle progression remain incompletely understood.

Toxin–antitoxin (TA) systems are widely encoded in *Mtb*, with as many as 88 putative modules, including an expanded repertoire of RelBE family members (Gerdes et al., 2005; Ramage et al., 2009; Slayden et al., 2018). Traditionally associated with stress responses and persistence, their physiological roles in *Mtb* remain debated, with conflicting evidence from overexpression and deletion studies (Goormaghtigh et al., 2018; Gupta et al., 2017; LeRoux et al., 2020; Pu et al., 2025). These inconsistencies suggest that individual TA loci, within the systems, may not function as generalized stress-response elements but instead are coordinated and operate within more defined physiological contexts. The RelBE1 system (*rv1246c–rv1247c*) is uniquely positioned adjacent to *rv1248c*, encoding α-ketoglutarate decarboxylase (Kgd), a key enzyme in an alternative branch of the TCA cycle active under nutrient-limited and lipid-rich conditions (Slayden et al., 2013, 2018). Similarly, the cell division regulator *madR1* (*rv2216*) is co-localized with *dlaT* (*rv2215*), encoding the E2 component of pyruvate dehydrogenase (Crew et al., 2015). These genomic arrangements suggest transcriptional and functional coupling between regulatory modules and central metabolic nodes (Figure 1). Under host-relevant conditions, where *Mtb* transitions from glycolytic to lipid-based carbon sources, such coupling may enable coordinated control of metabolism and growth.

We propose that RelBE1 and MadR1 function as genomically encoded metabolic control modules that couple carbon source availability to cell division, growth state, and persistence-associated physiology. In this framework, environmental inputs, particularly carbon source availability, are integrated through transcriptional and translational processes linked to central metabolic pathways. The genomic organization of *relBE1* adjacent to *kgd* and *madR1* co-localized with *dlaT* suggests potential transcriptional coupling between these regulatory modules and key nodes of the tricarboxylic acid (TCA) cycle. RelBE1, through post-transcriptional modulation of translation, and MadR1, through regulation of cell morphology and cell-cycle-associated processes, may therefore function as complementary regulators associated with metabolic state, redox balance, and adaptation to host-imposed stress (Crew et al., 2015; Ramirez et al., 2013). Together, these relationships support a model in which metabolic adaptation and persistence are coordinated through integrated regulatory–metabolic networks embedded within central carbon metabolism. Importantly, the mechanistic basis linking carbon-state-dependent metabolism to RelBE1 and MadR1 activity remains incompletely defined and is discussed here as a hypothesis-generating framework.

As a Perspective, this article integrates published observations with hypothesis-generating interpretations intended to stimulate experimental investigation into metabolic-regulatory coupling during persistence. This perspective examines the role of RelBE1 and MadR1 in carbon-dependent metabolic adaptation of *Mtb*. By reframing TA systems as coordinated metabolic integrators rather than generalized stress-response modules, we propose a model in which regulatory–metabolic coupling contributes to persistence-associated physiology. The genomic organization of *relBE1* and *madR1* suggests potential functional coupling between these regulatory modules and central carbon metabolism under host-relevant conditions. Together, these concepts provide a mechanistic basis for understanding how carbon-state-dependent regulation drives persistence in *Mtb* (Table 1).

**TABLE 1:** Reframing toxin-antitoxin systems: from stress modules to metabolic regulators.

Feature	Conventional view	Expanded view (this perspective)
Primary role	Stress survival, post-segregational killing	Carbon-state-dependent metabolic regulation and persistence control
Activation context	General stress (starvation, antibiotics)	Defined nutrient shifts (e.g., glucose → fatty acids/cholesterol)
Regulatory level	Growth arrest via toxin activation	Integrated control at translational (RelBE1) and transcriptional (MadR1-linked systems) levels
Molecular function	Global translation inhibition	Selective translational gating aligned with metabolic flux
Genomic organization	Isolated TA operons	Physically coupled to central metabolic enzymes (e.g., Kgd, DlaT)
Physiological output	Dormancy or slowed growth	Coordinated metabolic rerouting, redox balance, and persistence adaptation
Systems interpretation	Stress-response module	Integrated regulatory–metabolic subroutine (“survival algorithm”)
Therapeutic implication	Limited due to redundancy	Targetable carbon-state-dependent vulnerability during persistence

## Expanded mechanism of regulation for TA systems in *M. tuberculosis*: beyond protein-protein interaction

Toxin–antitoxin (TA) systems were initially described as plasmid maintenance modules but are now recognized as chromosomally encoded regulators associated with stress adaptation, persistence, and metabolic remodeling (Gerdes et al., 2005; Page and Peti, 2016; Slayden et al., 2018). In *Mtb*, their precise physiological roles remain unresolved, with increasing evidence suggesting context-dependent regulatory functions rather than generalized stress responses. RelBE1 is a type II TA system in which the RelE toxin acts as a ribosome-dependent RNase that modulates translation, while the RelB antitoxin represses transcription through conditional cooperativity (Christensen et al., 2001; Hayes and Sauer, 2003; Overgaard et al., 2008; Slayden et al., 2018). Beyond this canonical model, regulation of RelBE systems in *Mtb* is further expanded by the Crp-dependent antisense RNA asRelE2, which regulates RelE2 in *cis* and raises the possibility that antisense-mediated regulation may coordinate activity among related TA modules in response to cellular bioenergetic state (Dawson et al., 2022). These features support a model in which TA systems function as localized post-transcriptional regulators that couple environmental and metabolic signals to translational output, reshaping proteome composition in response to metabolic cues (Figure 1). Such coupling extends the TA function beyond growth arrest, positioning these systems as regulators of metabolic state.

In this framework, RelBE family members act not as global translation inhibitors but as conditional modulators that reshape proteome composition in response to environmental and physiological cues (Table 1). This perspective aligns TA systems with other regulatory networks that integrate environmental sensing with metabolic control, supporting a role in carbon-state-dependent adaptation. In the following sections, we examine RelBE1 and MadR1 as representative regulatory modules that link carbon source availability to metabolic reprogramming and cell cycle in *Mtb*.

## Genomic coupling of RelBE1 and MadR1 to central carbon metabolism

The tricarboxylic acid (TCA) cycle in *Mtb* integrates diverse carbon inputs, reflecting the organism’s metabolic flexibility (Chang and Guan, 2021; Ehrt and Rhee, 2013; Lee et al., 2013). As shown in Figure 1, carbohydrate-derived pyruvate and host-derived lipids converge at acetyl-CoA, a central node that determines carbon flux through the TCA cycle or adaptive bypass pathways. Under lipid-rich, host-relevant conditions, flux is redirected through pathways such as the glyoxylate shunt to support persistence in carbohydrate-limited environments (Lee et al., 2013; McKinney et al., 2000; Muñoz-Elías and McKinney, 2005).

Beyond enzymatic control, genomic organization suggests an additional layer of regulation linking metabolism to growth and adaptation. The operonic arrangement of *dlaT* with *madR1* couples a SulA-like cell division regulator (MadR1; Rv2216) to the E2 component of pyruvate dehydrogenase (DlaT; Rv2215), positioning MadR1 to coordinate transcriptional and morphological responses with carbon entry through acetyl-CoA (Crew et al., 2015). Similarly, the *relBE1* operon (*rv*1246c–*rv*1247c) is located immediately upstream of *kgd* (*rv*1248c), encoding α-ketoglutarate decarboxylase, a key enzyme in an alternative TCA branch active under nutrient-limited and lipid-rich conditions (Slayden et al., 2013).

These genomic linkages suggest possible direct transcriptional and functional coupling between regulatory modules and central metabolic nodes. Coordinated expression of *relBE1* with *kgd* and *madR1* with *dlaT* suggests a possible mechanism for integrating carbon source availability into both translational and transcriptional regulation. Under host conditions characterized by reduced carbohydrate availability and increased reliance on lipids, such coupling may enable dynamic reprogramming of TCA cycle flux, redox balance, and growth state. This organization supports a model in which RelBE1 and MadR1 are embedded within central metabolism as carbon-state-responsive regulatory modules. By linking environmental inputs to key metabolic entry points and branch nodes, these systems provide a framework for coordinating metabolic adaptation with persistence-associated physiology.

## Nutrient availability- and carbon-source-responsive regulation of RelBE1 and MadR1

Host infection imposes dynamic metabolic constraints on *Mtb*, leading to significant shifts in the pathogen’s available carbon sources throughout the acute and chronic phases of disease. Early in infection, *Mtb* can utilize glucose and glycerol. As infection progresses and granulomas mature, carbohydrate levels decline while host-derived lipids, including cholesterol and long-chain fatty acids, become enriched (de Carvalho et al., 2010; Peyron et al., 2008). These changes necessitate significant remodeling of central carbon metabolism, including redirection of TCA cycle flux, activation of the glyoxylate shunt, and reliance on alternative anaplerotic inputs to sustain energy and redox balance (Figure 1).

Exposure to cholesterol and fatty acids induces extensive remodeling of central carbon metabolism and lipid utilization pathways in *Mtb*, including altered expression of glyoxylate and related metabolic functions (Chen et al., 2024; Pawełczyk et al., 2021). Operon prediction analyses and genomic organization suggest that *relBE1* and *kgd* may be co-transcribed under at least some conditions (Pelly et al., 2016), although direct experimental validation under host-relevant metabolic states remains limited. Their activity may therefore be regulated through toxin-antitoxin complex dynamics or broader metabolic cues associated with altered TCA flux. MadR1’s functional coupling with *dlaT* links its regulatory activity to pyruvate dehydrogenase-associated metabolism and carbon-state adaptation (Crew et al., 2015). Together, these observations support a model in which RelBE1 and MadR1 contribute to coordinated metabolic adaptation during host-imposed nutrient stress. Together, these observations support a model of a network of carbon-source-responsive regulators, including RelBE1 and MadR1, that act at the transcriptional and translational levels to modulate TCA activity and downstream metabolic outcomes (Figure 1). Together, these relationships support a model in which RelBE1 and MadR1 are embedded within carbon-source-sensing circuits that may influence TCA cycle routing and metabolic output.

## Functional interplay and cooperative regulation of MadR1 and TA modules

RelBE1 and MadR1 enable coordinated regulation across complementary layers: RelBE1 functions post-transcriptionally to regulate translation of adaptation-related transcripts, while MadR1 coordinates cell cycle progression with transcriptional remodeling of metabolic programs, including lipid catabolism (Slayden et al., 2018). As available carbon sources shift during infection from carbohydrates to host-derived lipids, the TCA cycle responds to maintain redox balance and survival (Ehrt et al., 2018). RelBE1 may sharpen or refine this shift, enforcing conditional translation arrest or selective transcript degradation to rebalance flux through *kgd*-dependent or glyoxylate routes (Figure 1).

Coordination of TA modules, including RelBE2 and RelBE3, likely supports this phenotypic plasticity. Rather than acting redundantly, these systems may partition function across different metabolic states. Identifying an antisense (as)RNA targeting RelE2, which is involved in the *cis*-regulation of the RelBE2 TA module in a Crp_*mtb*_-dependent manner, points to a network of regulators linked to more distinct metabolic cues (Dawson et al., 2022). The coordination of MadR1, TA modules, and carbon flux regulation enhances *Mtb’s* ability to respond to fluctuating host nutrient conditions. By aligning cell cycle progression and TA activity with the metabolic response of persistence, *Mtb* achieves durable homeostasis in the face of immune pressures, hypoxia, and starvation. While RelBE1 and MadR1 may act under distinct regulatory regimes, the evidence for coordinated activity raises an important question: Are these modules independently responsive to overlapping stress cues, or do they function as components of an integrated persistence network? The genomic proximity of each regulator to a TCA cycle enzyme suggests possible co-transcription or co-regulation, yet their mechanistic roles, RelBE1 at the translational level and MadR1 at the transcriptional and morphological level, indicate potentially complementary rather than redundant functions (Korch et al., 2015). Dissecting whether these systems operate in parallel, sequentially, or within a larger co-regulated metabolic logic network is essential to understanding how *Mtb* layers control across regulatory hierarchies. These integrated regulatory layers represent potential targets for disrupting *Mtb* persistence by uncoupling translation control from carbon metabolism.

## Mechanistic and therapeutic implications

The genomic positioning of *relBE1* and *madR1* suggests that these modules are not isolated stress-response elements but are core components of an integrated carbon-state-responsive metabolic regulatory architecture. The *relBE1* locus is genomically integrated with *kgd*, which encodes α-ketoglutarate decarboxylase, while *madR1* is encoded in pairs with *dlaT*, which encodes a subunit of pyruvate dehydrogenase. This genomic organization supports the idea that *Mtb* has evolved a genetically encoded regulatory architecture to couple nutrient sensing with metabolic control at both transcriptional and translational levels.

During host infection, *Mtb* must balance redox homeostasis and biosynthetic demands as it transitions from glycolytic to lipid-based carbon sources such as cholesterol and fatty acids (de Carvalho et al., 2010; Peyron et al., 2008). These substrates alter carbon flux through the TCA cycle and engage adaptive pathways, including the glyoxylate and methylcitrate cycles, which are important for survival under hypoxic and nutrient-limited conditions (McKinney et al., 2000), ensuring that growth and metabolism are tuned to carbon flux through the TCA cycle and to prevailing nutrient conditions (Ehrt et al., 2018). In this context, RelBE1 and MadR1 may function as conditionally engaged regulatory modules that coordinate metabolic adaptation with growth control. RelBE1 may contribute to selective translational modulation under defined metabolic conditions, whereas MadR1 may integrate metabolic state with transcriptional and morphological responses linked to carbon utilization. Together, these systems may help maintain metabolic balance and resource allocation during persistence-associated stress, particularly in hypoxic, lipid-rich host environments.

From a therapeutic perspective, these findings highlight RelBE1 and MadR1, along with related pathways, as promising targets for intervention strategies to disrupt metabolic homeostasis during persistent infection. Targeting MadR1 or TA systems may reveal latent metabolic vulnerabilities masked during active replication but become critical under dormancy-inducing stress. Such insights could inform the development of persistence-directed therapies–an urgent need given the prolonged treatment times and relapse rates associated with TB. In this context, coupled mechanisms such as MadR1 and TA systems should be viewed not as passive stress responders, but as active drivers of metabolic plasticity. Their tight coupling to carbon flux redefines them as central components of the *Mtb* persistence program, positioned at the intersection of environmental sensing, translational control, and metabolic adaptation.

## Discussion

Despite increasing evidence that RelBE1 and MadR1 contribute to carbon-responsive regulation of metabolism in *Mycobacterium tuberculosis*, the mechanistic basis of this coupling remains incompletely defined. Their genomic proximity to key metabolic enzymes, including Kgd and DlaT, supports a model in which regulatory modules are physically and functionally integrated with central carbon metabolism. However, the extent to which this organization translates into coordinated regulation under host-imposed conditions remains an open question (Ehrt and Rhee, 2013; Ehrt et al., 2018).

A central unresolved issue is the specificity of RelE1-mediated translational control. While RelE toxins are known to cleave mRNAs in the ribosomal A-site and globally inhibit translation under stress (Christensen et al., 2001; Hayes and Sauer, 2003), it is not known whether RelE1 selectively targets transcripts associated with particular metabolic states. RelE-family toxins function as ribosome-dependent endonucleases that cleave mRNA within the ribosomal A-site during stress adaptation (Gerdes et al., 2005). Structural and biochemical studies in *Escherichia coli* and *Thermus thermophilus* demonstrated that RelE binding induces conformational distortion of the A-site codon and promotes site-specific cleavage through ribosome-assisted hydrolysis rather than through a classical catalytic triad (Neubauer et al., 2009). Early *in vitro* studies suggested codon-specific cleavage preferences, whereas subsequent *in vivo* studies demonstrated broader translational shutdown across actively translated transcripts (Pedersen et al., 2003). Nevertheless, several studies identified reproducible sequence biases, including preferential cleavage near Py-Pu-G motifs, indicating that RelE activity may not be entirely indiscriminate. Comparative analyses of RelE homologs across bacterial phyla further demonstrated “relaxed specificity,” in which ribosome dependence is conserved but cleavage preference and kinetics vary among homologs. In mycobacteria, work from Korch and Clark-Curtiss established that RelBE-family loci are stress-responsive and expressed during macrophage infection, supporting the broader concept that TA activity may be coordinated with defined physiological or metabolic states (Korch et al., 2015). Accordingly, we propose that RelE1 may contribute to the conditional reshaping of proteome composition under specific metabolic environments; however, the mechanisms underlying direct transcript selectivity and physiological specificity remain unresolved and require experimental validation. We therefore propose that RelE1 may preferentially reshape proteome composition under defined metabolic conditions rather than indiscriminately induce translational arrest. However, this remains speculative and requires direct experimental testing. This concept is consistent with emerging views that persistence involves controlled metabolic downscaling rather than complete growth arrest (Ehrt et al., 2018; Samuels et al., 2022). Defining the substrate specificity of RelE1 under physiologically relevant conditions will be essential for determining whether TA systems act as global inhibitors or selective regulators of proteome composition.

Given that lipid utilization and redox balance are tightly coupled during intracellular growth (de Carvalho et al., 2010; Marrero et al., 2010), determining whether MadR1 responds directly to carbon source availability, redox state, or upstream regulatory pathways will be important for defining its role in coordinating growth and metabolism. Prior work demonstrated that *madR1* (*rv2216*) is functional within a polycistronic operon with *dlaT* (*rv2215*), *lipB*, and *lipA*, linking MadR1 to pyruvate dehydrogenase-associated metabolism and lipoate-dependent metabolic functions (Crew et al., 2015). In addition, MadR1 overexpression induced transcriptional programs associated with lipid β-oxidation, secondary metabolism, dormancy-associated lipid remodeling, and alternative respiratory adaptation (Crew et al., 2015). These observations support a functional association between MadR1 and metabolic adaptation during host-relevant stress conditions. However, the upstream mechanisms linking carbon availability or metabolic state to *madR1* and *dlaT* regulation remain unresolved. In this Perspective, the phrase “responsive to TCA cycle flux” is therefore intended to reflect potential coupling between metabolic state, pyruvate dehydrogenase-associated metabolism, and regulatory activity rather than direct metabolite sensing by MadR1 itself.

An additional unresolved question is whether RelBE1 and MadR1 operate independently or as components of a coordinated regulatory network. Their genomic linkage to distinct nodes of central metabolism, *relBE1* to *kgd* and *madR1* to *dlaT*, suggests the potential for integrated control of both TCA cycle entry and downstream flux. However, their mechanistic roles differ: RelBE1 acts at the level of translation, while MadR1 influences transcriptional programs and cell morphology. This raises the possibility that these systems function in a layered manner, with transcriptional and translational regulation jointly shaping metabolic state under defined carbon conditions. Prior work demonstrated that *madR1* is encoded within a polycistronic operon with *dlaT*, *lipB*, and *lipA*, genetically linking MadR1 to pyruvate dehydrogenase-associated metabolism and lipoate-dependent metabolic regulation. In parallel, MadR1 overexpression induced pathways associated with lipid β-oxidation, lipid remodeling, and persistence-associated metabolic adaptation. Together with the genomic linkage of *relBE1* to *kgd*, these observations support the broader concept that regulatory modules may be functionally integrated with central carbon metabolism during host adaptation. However, the upstream mechanisms linking carbon source availability or TCA flux to *relBE1* and *madR1* expression remain undefined. Potential contributors may include redox-responsive transcriptional regulators, Crp-associated signaling pathways, energetic stress responses, or broader metabolic remodeling networks; however, these relationships remain hypothetical (Ehrt and Rhee, 2013; Slayden et al., 2013).

Testing this model will require experimental approaches that directly link regulatory activity to metabolic flux. For example, isotope tracing under defined carbon conditions could determine whether perturbation of RelBE1 or MadR1 alters carbon routing through the TCA cycle or associated bypass pathways (de Carvalho et al., 2010). Similarly, ribosome profiling and transcriptome analysis could reveal whether RelE1 activity selectively reshapes the proteome in a carbon-dependent manner. Genetic interaction studies, including conditional knockdown or combinatorial perturbation of *relBE1* and *madR1*, will be essential to determine whether these systems act in parallel, sequentially, or as components of an integrated regulatory module.

From a physiological perspective, the proposed coupling of regulatory modules to central metabolism suggests a mechanism by which *Mtb* maintains homeostasis under fluctuating nutrient conditions encountered during infection. Rather than inducing a uniform growth arrest, RelBE1 and MadR1 may enable graded modulation of metabolic activity, allowing the bacterium to balance energy production, redox state, and biosynthetic demand. This model aligns with the requirement for sustained, low-level metabolic activity during persistence, particularly in hypoxic and lipid-rich environments (Ehrt et al., 2018; Warner, 2014).

These observations support a broader conceptual shift in how TA systems are viewed in *Mtb*. Instead of functioning primarily as generalized stress-response elements (Page and Peti, 2016), RelBE1 and related modules may act as metabolic integrators that couple environmental inputs to physiological outputs by selectively regulating translation. In parallel, MadR1 provides a complementary layer of control that links metabolic state to cell division and cell cycle progression under alternative carbon sources. Together, these systems support a regulatory architecture in which environmental sensing, metabolic flux, and growth control are tightly coordinated.

This framework has important implications for therapeutic development. If RelBE1 and MadR1 are required for maintaining metabolic homeostasis under host-relevant conditions, then disrupting their function may expose vulnerabilities that are not apparent during active replication. Targeting such regulatory–metabolic coupling could provide a strategy to destabilize persistence-associated physiology and enhance the efficacy of existing antimicrobials. More broadly, this framework positions RelBE1 and MadR1 as core components of a carbon-state-dependent regulatory architecture, redefining toxin–antitoxin systems as active determinants of metabolic plasticity and persistence in *M. tuberculosis* (Figure 1 and Table 1).

## Data Availability

The original contributions presented in this study are included in this article/supplementary material, further inquiries can be directed to the corresponding author.
